# Developmental Formation of the GABAergic and Glycinergic Networks in the Mouse Spinal Cord

**DOI:** 10.3390/ijms23020834

**Published:** 2022-01-13

**Authors:** Chigusa Shimizu-Okabe, Shiori Kobayashi, Jeongtae Kim, Yoshinori Kosaka, Masanobu Sunagawa, Akihito Okabe, Chitoshi Takayama

**Affiliations:** 1 Department of Molecular Anatomy, Graduate School of Medicine, University of the Ryukyus, 207 Uehara, Nishihara 903-0215, Japan; chigusa@med.u-ryukyu.ac.jp (C.S.-O.); pop-american@piano.ocn.ne.jp (S.K.); hot_cola_0225@yahoo.co.jp (Y.K.); sho20000919@yahoo.co.jp (M.S.); 2 Department of Anatomy, Kosin University College of Medicine, Busan 49267, Korea; island7805@gmail.com; 3 Department of Nutritional Science, Faculty of Health and Welfare, Seinan Jo Gakuin University, Fukuoka 803-0835, Japan; okabe@seinan-jo.ac.jp

**Keywords:** astrocyte, gamma-aminobutyric acid (GABA), GABA transporter (GAT), GABA_A_ receptor, glutamic acid decarboxylase (GAD), glycine, glycine receptor, glycine transporter (GlyT), K^+^-Cl^−^ cotransporter 2 (KCC2), vesicular GABA transporter (VGAT)

## Abstract

Gamma-aminobutyric acid (GABA) and glycine act as inhibitory neurotransmitters. Three types of inhibitory neurons and terminals, GABAergic, GABA/glycine coreleasing, and glycinergic, are orchestrated in the spinal cord neural circuits and play critical roles in regulating pain, locomotive movement, and respiratory rhythms. In this study, we first describe GABAergic and glycinergic transmission and inhibitory networks, consisting of three types of terminals in the mature mouse spinal cord. Second, we describe the developmental formation of GABAergic and glycinergic networks, with a specific focus on the differentiation of neurons, formation of synapses, maturation of removal systems, and changes in their action. GABAergic and glycinergic neurons are derived from the same domains of the ventricular zone. Initially, GABAergic neurons are differentiated, and their axons form synapses. Some of these neurons remain GABAergic in lamina I and II. Many GABAergic neurons convert to a coreleasing state. The coreleasing neurons and terminals remain in the dorsal horn, whereas many ultimately become glycinergic in the ventral horn. During the development of terminals and the transformation from radial glia to astrocytes, GABA and glycine receptor subunit compositions markedly change, removal systems mature, and GABAergic and glycinergic action shifts from excitatory to inhibitory.

## 1. Introduction

In the mature central nervous system (CNS), which includes the spinal cord, γ-aminobutyric acid (GABA), and glycine, are inhibitory neurotransmitters that negatively regulate neuronal activity [1,2,3,4]. In the spinal cord, there are three types of inhibitory neurons and terminals: GABAergic, GABA/glycine coreleasing, and glycinergic [5,6,7]. These neurons and terminals are arranged in the spinal cord neural circuit and are involved in many vital roles, such as regulating somatic sense, locomotive movement, and respiratory rhythms [8,9,10]. In the first part of this review, we will focus on the three types of neurons and terminals and describe the inhibitory networks in the mature spinal cord from the following viewpoints: (1) distribution of neurons and terminals, (2) receptor composition, (3) removal system, and (4) mechanisms underlying inhibitory transmission. In the latter half of the review, we will focus on morphological development and demonstrate the processes of how mature networks are established through the following neuronal differentiation processes: GABAergic and glycinergic neurons are born in the ventricular zone, migrate in the gray matter, extend their dendrites and axons, and form synapses. During these processes, the neuronal types alter the composition of the receptor subunits changes, the removal system matures, and the action of both neurotransmitters shifts from excitatory to inhibitory.

## 2. GABAergic and Glycinergic Network in the Mature Spinal Cord

**Figure 1:**
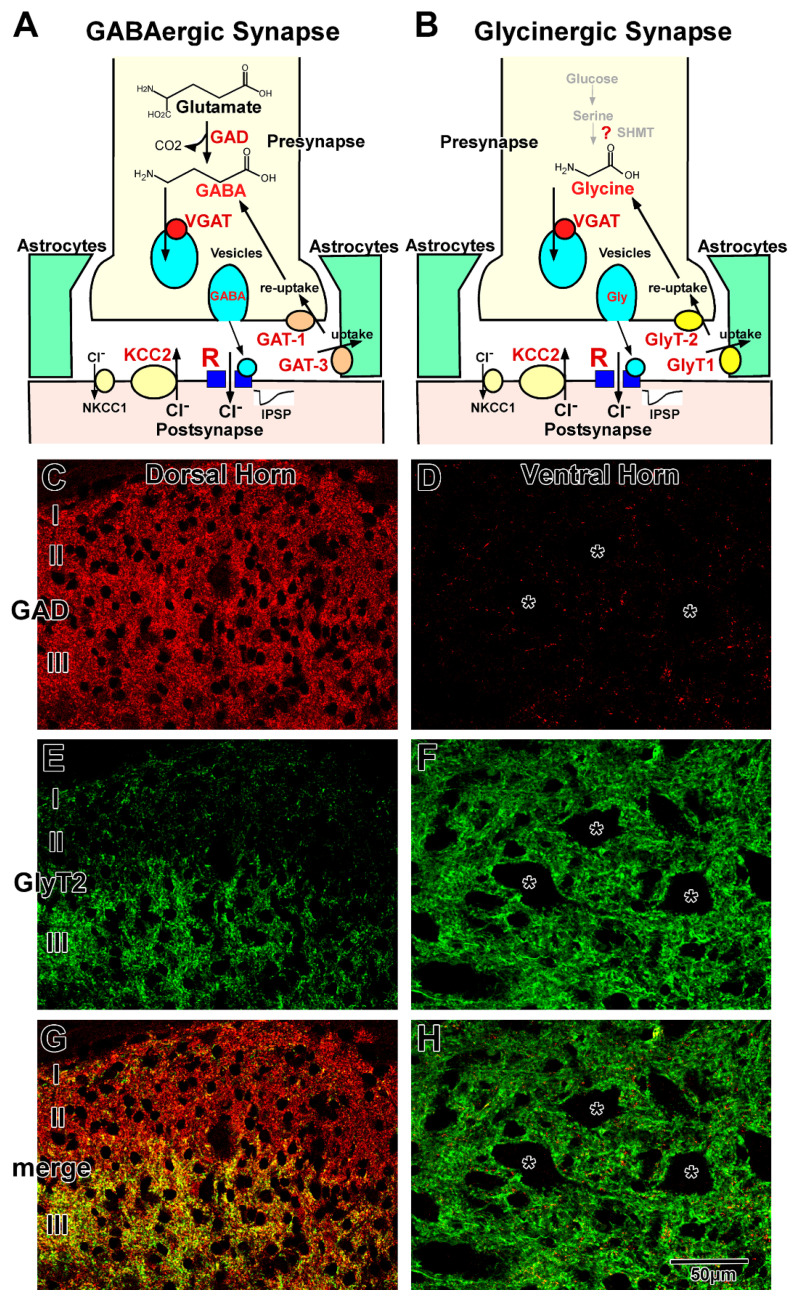
GABAergic and glycinergic transmission in the adult spinal cord. (**A**,**B**) are Schematic illustrations of GABAergic (**A**) and glycinergic (**B**) synapses. Various key molecules are involved in GABAergic and glycinergic transmission. C and D are Immunohistochemistry for GAD (**C**,**D**), GlyT2 (**E**,**F**), and both (**G**,**H**) in the dorsal (**C**,**E**,**G**) and ventral (**D**,**F**,**H**) horns. In lamina I and II, GABAergic terminals are dominant (**C**), whereas GAD and GlyT2 double-positive colocalizing terminals (yellow) are dominant in lamina III of the dorsal horn (**G**). In the ventral horn, glycinergic GlyT2-positive terminals (green) are dominant (**F**), but GABAergic GAD-positive terminals (red) are scarce (**F**,**H**).

GABAergic and glycinergic synapses are schematically illustrated in Figure 1A,B, respectively. GABA and glycine are synthesized within the neurons and transported from the extracellular space, including the synaptic cleft. The neurotransmitters are loaded into synaptic vesicles and released from the axon terminals. After diffusion in the synaptic cleft, they bind to GABA or glycine receptors at the postsynaptic membrane. In the mature spinal cord, activation of GABA or glycine receptors induces hyperpolarization of the membrane potential and negatively regulates neuronal activity. The action of these neurotransmitters is terminated by their removal from the synaptic cleft into presynaptic terminals and astrocytic sheets that surround the synapses [3,11,12,13].

### 2.1. GABAergic Transmission

GABAergic transmission at the synapse is illustrated in Figure 1A. GABA is synthesized from glutamate by two isoforms of glutamic acid decarboxylase (GAD65 and GAD67) [14]. The primary source of glutamate may be derived from glutamine, which is transported back from astrocytes by glutamine transporter [15]. GABA is loaded into vesicles by vesicular GABA transporter (VGAT), also known as vesicular inhibitory amino acid transporter (VIAAT) [16,17]. GABA is released via the fusion of vesicles with the presynaptic membrane at the nerve terminals and binds to GABA receptors on the postsynaptic membrane.

GABA receptors are classified into three groups based on their pharmacological and biochemical characteristics: GABA_A_, GABA_B_, and GABA_C_. Most of the fast synaptic transmission is mediated by GABA_A_ receptors in the mammalian CNS [1,18]. The GABA_A_ receptor is a member of the ligand-gated ion channel receptor family and is thought to be composed of five heteromeric subunits belonging to seven different subunit families: α1–6, β1–3, γ1–3, δ, ε, π, and θ [1,2,18,19]. Native GABA_A_ receptors contain at least one α, one β, and one γ subunit. The subunit composition varies among the brain regions [20,21,22]. Different subunit compositions exhibit their own characteristic pharmacological and electrophysiological properties [1,2,19,23,24]. The GABA_C_ receptor is also an ion-channel type receptor composed of only single or multiple ρ subunits: ρ1 and ρ2. The GABA_C_ receptor is identified as a bicuculline and baclofen insensitive GABA receptor and is considered a pharmacological variant of GABA_A_ receptors [18,25,26]. The binding of GABA to the GABA_A_ and GABA_C_ receptors induces the influx of chloride ions (Cl^−^) and mediate hyperpolarization of the postsynaptic membrane potential. The GABA_B_ receptor, which consists of two subunits, GABA_B1_ and GABA_B2_, is a metabotropic receptor, activates G proteins, negatively regulates the second messenger system, and responds to slow-acting inhibition of channel and receptor functions [27,28,29,30,31,32]. GABAergic transmission is terminated by the reuptake of GABA into the nerve terminals or uptake into the surrounding astrocytes by plasma membrane GABA transporters (GATs) [33].

GATs are high-affinity Na^+^ and Cl^−^-dependent transporters that cotransport GABA with Na^+^ and Cl^−^ [34,35]. In the CNS, there are three types of GATs: GAT-1, GAT-2, and GAT-3. GAT-2 is localized in leptomeningeal ependymal cells and the choroid plexus [36]. GAT-1 and GAT-3 function at the membranes of axon terminals containing GABAergic vesicles and astrocytic sheets surrounding GABAergic synapses, respectively [37,38,39,40]. In the astrocytes, GABA is degraded into succinate, followed by entering the Krebs cycle [15]. The 2-oxoglutarate in the Krebs cycle is converted to glutamate and glutamine. Astrocytic glutamine is transported back to neurons by a glutamine transporter. This GABA/glutamine cycle may play critical roles in GABA metabolism between neurons and astrocytes [41].

### 2.2. Glycinergic Transmission

Glycinergic transmission at the synapse is illustrated in Figure 1B. The metabolic pathway of glycine in neurons is still unclear. Although serine hydroxymethyltransferase (SHMT) has been shown to be able to synthesize in the neurons [42,43,44], little is known about the relationship between SHMT and glycine levels [45]. In general, high-affinity uptake systems, mediated by glycine transporter 2 (GlyT2), are considered the principal regulators of intracellular glycine concentrations. The GlyT2 knockout mice die from lack of glycine during the second postnatal week [46,47], which suggests that de novo synthesis by SHMT is not sufficient for glycinergic neurotransmission [4,12,48], and glycine in the neurons may be dominantly transported from extracellular space through blood–brain barrier. Thus, GlyT2 is a reliable marker for glycine-immunoreactive neurons [45]. After being loaded into vesicles via VGAT (VIAAT) [16,17], glycine is released by exocytosis from the nerve terminals and binds to glycine receptors on the postsynaptic membrane. The glycine receptor is a ligand-gated ion channel receptor that consists of five subunits belonging to two subunit families: α1–3 and β in the mammalian CNS [3,49,50]. The α subunit has a strychnine binding site, and the β subunit binds to the scaffolding protein gephyrin. The composition varies among the CNS regions. Different subunit compositions exhibit their own characteristic electrophysiological properties [50,51]. Glycine binding to the receptor induces an influx of Cl^−^ (Figure 1A), as observed in GABA binding (Figure 1B). The glycinergic action is terminated by reuptake into the nerve terminals and uptake into the surrounding glia through plasma membrane glycine transporters (GlyTs) [52]. GlyTs are high-affinity Na^+^ and Cl^−^-dependent transporters that co-transport glycine with Na^+^ and Cl^−^. There are two types of GlyTs in the CNS: GlyT1 and GlyT2. In the spinal cord, as well as in other brain regions, GlyT1 is localized at the astrocytic sheets that surround glycinergic synapses, and GlyT2 is localized at the membranes of axon terminals that contain glycinergic vesicles [53,54]. In the astrocyte surrounding glycinergic synapses, glycine may be degraded by the glycine cleavage system [55,56].

### 2.3. GABAergic and Glycinergic Transmission in the Mature Spinal Cord

GABA or GAD immunohistochemistry [57,58,59] and GAD-green fluorescent protein (GFP) labeling [60] demonstrated that the density of GABAergic neurons is high in the dorsal horn and moderate in the middle part of the gray matter central part, whereas GABAergic neurons are scarce or in the ventral horn. The distribution of GABAergic terminals is almost the same as that of GABAergic neurons, with high density observed in the dorsal horn (Figure 1C) and low density observed in the ventral horn (Figure 1D). In contrast, glycine immunohistochemistry [61] and GlyT2 expression analysis [8,10] demonstrated that glycinergic neurons and terminals are homogeneously distributed in the gray matter, except for in the superficial layer of the dorsal horn (Figure 1E,F). In lamina I and II, the density of glycinergic neurons and terminals was lower than that observed in the other laminae (Figure 1E) [61,62]. Furthermore, double staining of GABA/GAD and glycine/GlyT2 demonstrated that colocalizing (functionally coreleasing) neurons and terminals are often detected in the spinal cord (Figure 1G,H) [5,6,7], and electrophysiological studies confirmed that the two neurotransmitters are loaded into the same synaptic vesicles and released simultaneously [63,64]. Coreleasing terminals have been abundantly detected in the deep part of the dorsal horn and middle part of the gray matter. In general, GABAergic neurons and their terminals are dominant in lamina I and II (Figure 1G). Coreleasing neurons and terminals are dominant in lamina III to VI (Figure 1G). Glycinergic neurons and their terminals are dominant in the lamina VII–IX (Figure 1H) [9,53,65,66,67]. Total inhibitory terminals detected as VGAT (VIAAT)-positive dots are homogeneously and ubiquitously distributed in the gray matter [57]. GABA and glycine play distinct roles in motor and sensory functions in the complex network in the dorsal [68,69] and ventral horns [70]. Fast GABAergic transmission is dominantly mediated by GABA_A_ receptors consisting of α3β3γ2 subunits in the dorsal horn and α2(α5)β3γ2 subunits in the motor neurons [21,71]. Glycinergic transmission is mediated by glycine receptors consisting of α1β (two α1 and three β) subunits [51,72]. GABA_A_ and glycine receptors colocalize at the postsynaptic membrane of the coreleasing terminals [73]. GABA_B_ Receptors consisting of B1, 1a and 1b, and B2 mainly play roles in the dorsal horn and motor neuron pool [74,75]. [3H]-baclofen binding assay demonstrated that GABA_B_ receptor activity may be higher in the dorsal horn than in other areas [74]. GABAc receptors, containing only ρ2 subunits, play roles in the dorsal horn, whereas motor neurons express those consisting of both ρ1 and ρ2 subunits [76]. Released GABA is removed into presynaptic terminals by GAT-1 and into astrocytic sheets by GAT-3. In contrast, uptake of released glycine into the astrocytic processes is mediated by GlyT1, and reuptake of glycine into the presynaptic terminals is mediated by GlyT2. Because GAT-1 distribution is identical to that of the GABAergic terminals, GAT-1 is abundantly localized in the dorsal horn and sparsely localized in the ventral horn [77]. In contrast, GlyT2 is homogeneously distributed throughout the gray matter [53,78]. Although the localizations of GABAergic and glycinergic terminals are different, both GAT-3 and GlyT1 are homogeneously distributed throughout the astrocytic sheets surrounding synapses, suggesting that astrocytic uptake may ubiquitously occur regardless of terminal distribution.

### 2.4. Regulation of GABAergic and Glycinergic Action by Chloride Transporters

**Figure 2:**
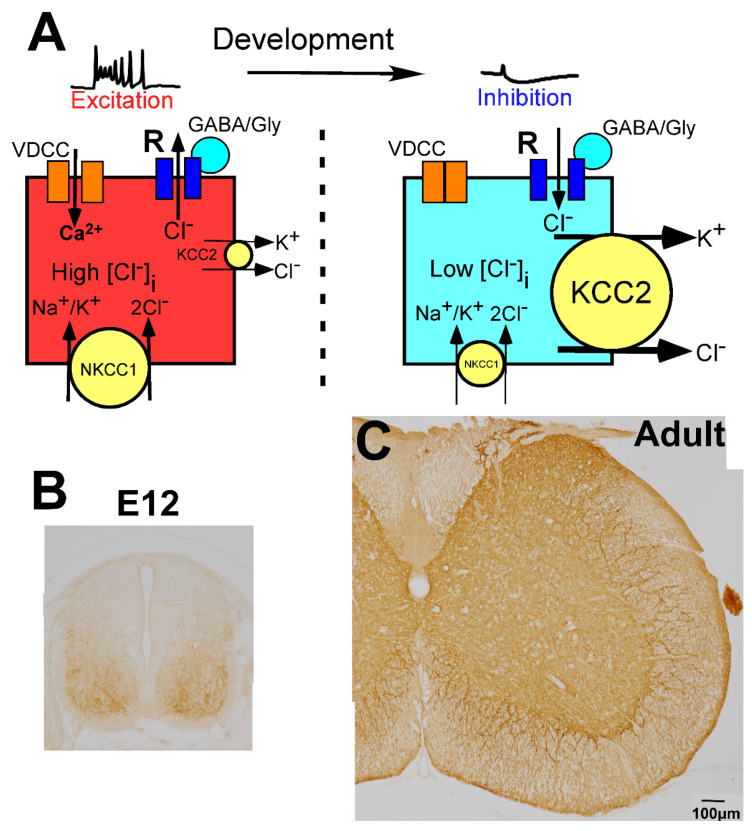
Molecular mechanisms underlying developmental changes in the action of GABA and glycine. (**A**) Schematic illustration of GABA action depending on intracellular Cl^−^ concentration ([Cl^−^]i), which is regulated by K^+^, Cl^−^ cotransporter 2 (KCC2), and Na^+^, K^+^, Cl^−^ cotransporter 1 (NKCC1). In the immature stage, KCC2 expression is low and [Cl^−^]i is high, thus, GABA binding to GABA receptors (R) induces the efflux of chloride ions (Cl^−^) and mediates excitation (left). In contrast, after maturation, KCC2 expression is high and [Cl^−^]i is low, thus, GABA mediates inhibition. When GABA/glycine elevates the membrane potential in the immature spinal cord, Ca^2+^ enters through the activated voltage-dependent calcium channel (VDCC) (left). (**B**,**C**) Developmental localization of KCC2. KCC2 is weakly localized in the ventral horn at E12, but the dorsal part was negative (**B**). In the adult spinal cord, KCC2 is expressed throughout the gray matter (**C**).

In the CNS, the change in membrane potential exerted by GABA and glycine is determined by the intracellular chloride ion concentration ([Cl^−^]_i_), which is regulated by the balance of two different chloride cotransporters, Na^+^-K^+^-Cl^−^ cotransporter 1 (NKCC1) and K^+^-Cl^−^ cotransporter 2 (KCC2) (Figure 2A) [79,80,81]. NKCC1 increases the [Cl^−^]_i_, whereas KCC2 decreases the [Cl^−^]_i_. When the action of NKCC1 is relatively high or KCC2 is absent, [Cl^−^]_i_ is high, and GABA and glycine induce depolarization of the membrane potential (Figure 2A, left). In contrast, when KCC2 expression is high compared with the expression of NKCC1, [Cl^−^]_i_ is low, and GABA and glycine act in an inhibitory fashion (Figure 2A, right). In the mature CNS, which includes the spinal cord, KCC2 is highly expressed on the membranes of neuronal cell bodies and dendrites (Figure 2C) [57], and its expression level is very high compared with that of NKCC1 [79,80,81]. Thus, in the mature spinal cord, the [Cl^−^]_i_ is low enough for GABA and glycine to act as inhibitory neurotransmitters [57,82].

## 3. Development of GABAergic and Glycinergic Neurons and Their Axon Terminals

In general, spinal cord development progresses in the ventral-to-dorsal and rostral-to-caudal directions [83,84]. The formation of synapses, maturation of the removal system, and changes in the action of GABA and glycine also proceed in the same directions. This ventral-to-dorsal development may be regulated by sonic hedgehog signals [85,86,87].

### 3.1. Early Development of the GABAergic and Glycinergic Neurons

**Figure 3:**
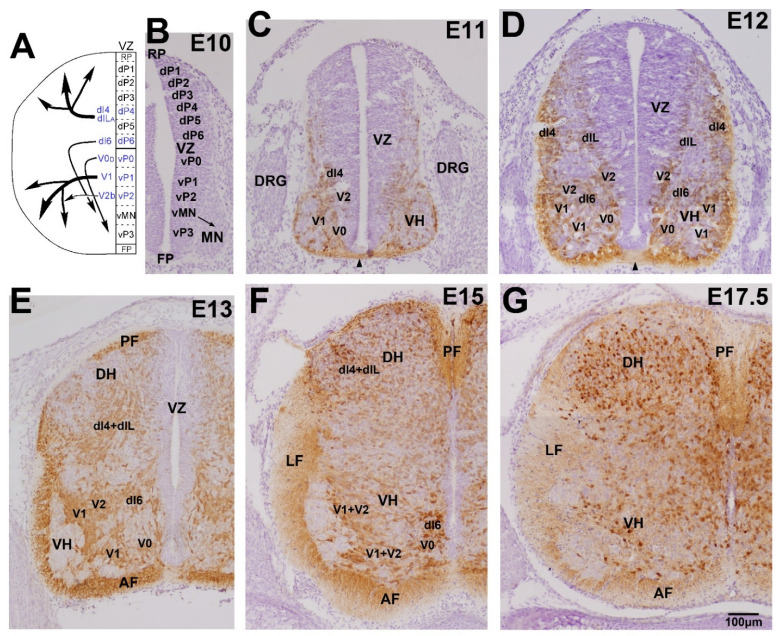
Developmental localization of GABAergic neurons in the spinal cord. (**A**) Schematic illustration of the origin of GABAergic neurons and their migration routes. GABAergic neurons are derived from six classes (V0D, V1, V2b, dI6, dI4, and dIL) that arise from five domains (vP0, vP1, vP2, dP6, and dP4). Each class of GABAergic neurons migrates their own routes and settle in distinct laminae. (**B**–**G**) Immunohistochemical analysis of GFP in the developing GFP-GAD knock-in mouse spinal cord. GABAergic neurons were absent at E10 (**B**) and appeared at E11 (**B**), and expanded their localization during embryonic development in the ventral-to-dorsal direction (**D**–**G**). In contrast to the mature spinal cord (Figure 1B), many GABAergic neurons were detected homogeneously in the dorsal and ventral horn of the embryonic spinal cord.

The early development of GABAergic neurons has been precisely described in previous reviews [70,88,89]. Twelve progenitor domains are formed in the spinal cord ventricular zone (neuroepithelial layer), including the vMN and vP0–vP3 domains in the ventral half and the dP1–dP6 domains in the dorsal half (Figure 3A) [83,84]. Each domain produces distinct neuron groups (classes): MN and V0–V3 in the basal plate (ventral half) and dI1–dI6 in the alar plate (dorsal half). The dIL_A_ and dIL_B_ classes are derived later from the dP4 and dP5 domains. The V0 class is further divided into V0_V_ and V0_D_ subclasses, and the V2 class is subdivided into V2a and V2b subclasses. These classes are characterized by the expression of marker proteins such as Isl1, Evx1/2, and Evx1 [90]. GABAergic neurons are derived from six of the classes (V0_D_, V1, V2b, dI4, dI6, and dIL_A_). After exiting the cell cycle, neurons move out of the ventricular zone and migrate into the gray matter (Figure 3A). Figure 3 demonstrates the developmental localization of GABAergic neurons in embryonic heterozygous GAD-GFP knock-in mice [60]. In the cervical spinal cord, GABAergic neurons first appear on the surface of the ventricular zone between embryonic day 10 (E10) and E11 (Figure 3B,C). The neurons migrate along distinct routes and finally settle in distinct laminae (Figure 3A). The differentiation processes of GABAergic neurons have been precisely demonstrated using GAD or GABA immunohistochemistry [57,91,92] and GAD-GFP knock-in mice (Figure 3B–G) [60,93]. In contrast, the early developmental processes of glycinergic neurons are still unclear. RNA sequencing studies demonstrated that GlyT2 mRNA-expressing neuron groups were identical to those expressing GAD mRNA [94,95]. GlyT2-GFP histochemistry and glycine immunohistochemistry demonstrated that glycinergic neurons often colocalize with GABA or GAD after E13, suggesting that late embryonic development of glycinergic neurons may be the same as that of GABAergic neurons [62,96]. Taken together, these findings indicate that glycinergic neurons may be derived from the same domains as GABAergic neurons and may appear later than GABAergic neurons.

### 3.2. Development in the Ventral Horn

After motor neurons are differentiated from the MN class in the ventral horn, three classes of cells—V0_D_, V1, and V2b—develop into GABA and glycinergic neurons under the regulation of various transcriptional factors [70,97] and distinctly participate in the complex network around the motor neurons (Figure 3B–G) [98]. In the cervical spinal cord [57], V0_D_ neurons first appear on the ventral side of the sulcus limitans between E10 and E11. Subsequently, these cells move ventrally and send commissural axons into the contralateral marginal zone (Figure 3C,D). The axons ascend two to four segments and enter the contralateral motor neuron pool [57,70,99,100]. Finally, they give rise to inhibitory neurons in the mature lamina VII. Second, V1 neurons appear before E11, located ventrally to the V0 neurons, and V2b neurons subsequently arise between E12 and E13 (Figure 3C,D). These neurons settle in the ventral horn and extend their axons into the ipsilateral marginal zone. Their axons ascend or descend for several segments [101,102,103]. The V1 neurons develop into the major inhibitory interneurons, including Renshaw cells, in the ventral horn [103]. Inhibitory neurons derived from V2b are low in number among the neurons in lamina VII [104]. Last, the dl6 neurons derived from the dP6 domain migrate in the ventral direction into lamina VII and VIII and take part in the ventral horn network (Figure 3D,E). Glycinergic neurons appear at E13 in the ventral horn [62] when GlyT2 is localized at the axon varicosities in the marginal zone [53]. Double labeling of GABA and glycine demonstrated that glycine-positive neurons often contained GABA immunolabeling during embryonic development [62]. These results suggest that many GABAergic neurons may gradually convert to coreleasing neurons in the ventral horn after E13.

**Figure 4:**
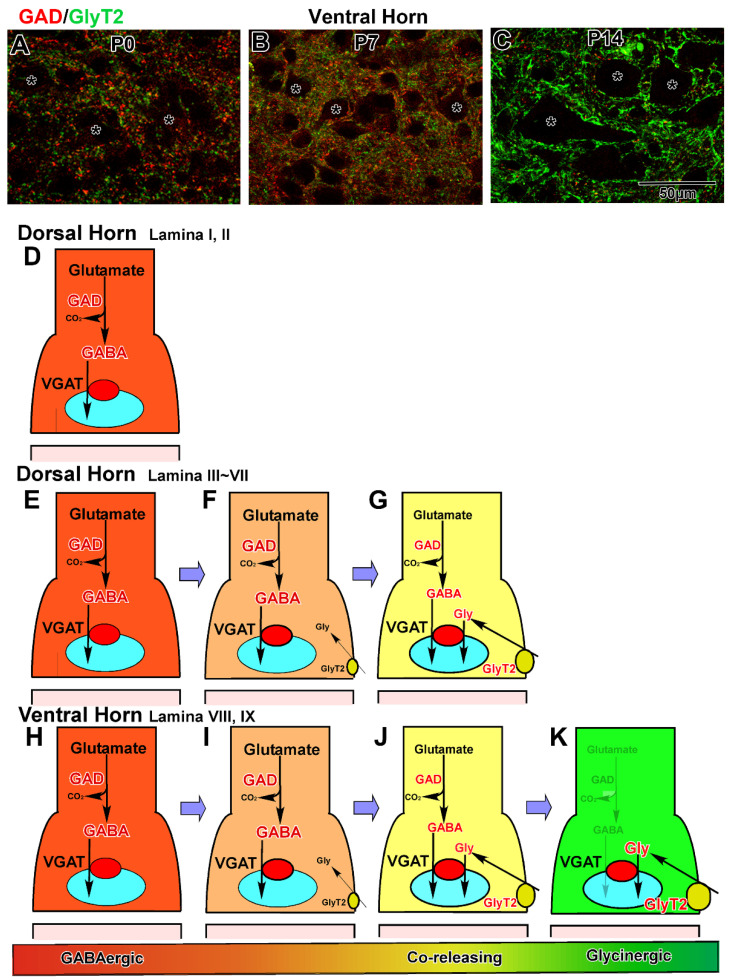
Developmental changes in inhibitory terminals in the spinal cord. (**A**–**C**) Double labeling of GAD and GlyT2 in the developing ventral horn. Dominant inhibitory terminals are GABAergic (red) at P0 (**A**) and change to coreleasing terminals at P7 (**B**) and glycinergic terminals at P14 (**C**). (**D**–**K**) Schematic illustrations of the developmental changes in inhibitory terminals. Initially, GABAergic terminals are formed after starting GABA synthesis by GAD in the gray matter (**D**,**E**,**H**). In lamina I and II, GABAergic terminals remain (**D**). In lamina III to IX, GABAergic terminals convert to coreleasing terminals by the additional glycine reuptake by GlyT2 (**F**,**G**,**I**,**J**). In lamina VIII and IX, GAD disappears from the terminals, and the coreleasing terminals give rise to glycinergic terminals (**K**).

In the ventral horn, GABAergic axon terminals, identified as axon varicosities, first appear in the marginal zone at E11 and are detected within the ventral horn at E13. They markedly increase in number and density after E15 and often surround the cell bodies of large motor neurons at E17 [57]. Until postnatal day seven (P7), they continue to increase in density markedly, and the neuropil region is occupied by numerous GABAergic terminals. In contrast, glycinergic terminals appear in the ventral horn at E15 and continue to increase during embryonic and postnatal development [53]. Double staining of GAD and GlyT2 revealed that GlyT2 immunolabeling was usually localized at GABAergic axon terminals, while GlyT2 single-positive terminals were scarce during embryonic development (Figure 4A). During postnatal development, GABAergic terminals gradually convert to coreleasing terminals in the marginal zone after E14 and in the ventral horn after E16. The coreleasing terminals markedly increase in density during the first postnatal week, and coreleasing terminals become dominant at P7 (Figure 4B). Between P7 and P14, the majority of coreleasing terminals change to glycinergic terminals via the removal of GAD from the terminals (Figure 4C). After P21, glycinergic terminals, detected as GlyT2-positive dots, often surround the large neurons, whereas GABAergic terminals are sparse in the ventral horn [53]. The aforementioned shift in dominant neurotransmitters may underlie the difference in the survival period of GAD67-knockout mice and GlyT2-knockout mice [46,47,52,105,106,107]. Because GABAergic inhibition is dominant at birth, GAD67-knockout mice cannot survive after birth, whereas GlyT2-knockout mice can. In contrast, GlyT2-knockout mice suffer from the neuromotor disorder and die around P10 because the dominant inhibitory input shifts from GABAergic to glycinergic during the second postnatal week.

### 3.3. Development in the Dorsal Horn

The dI4 class and late-born dIL_A_ subclass, derived from the dP4 domain, differentiate into GABAergic neurons in the dorsal horn (Figure 3A) [70,88,89]. GABAergic neurons appear on the surface of the dorsal ventricular zone between E11 and E13, migrate laterally and dorsally, and enter the dorsal horn after E13 (Figure 3C–E) [57]. The number of GABAergic neurons markedly increases until E17, and the neurons distribute throughout lamina I to IV by the day of birth (Figure 3E–G). Glycinergic neurons may be derived from the same dorsal domains under the direction of transcription factors such as Ptfla [89,95]. Double staining with GAD/GABA and GlyT2/glycine suggests that GABAergic neurons in lamina I through IV may remain GABAergic before birth [53,62,96]. The GABAergic neurons convert to coreleasing neurons in lamina IV and lamina III during postnatal development, whereas many GABAergic neurons remain GABAergic in lamina I and II until maturation is complete.

GABAergic axon terminals are first detected in the dorsal horn at E15, markedly increase in density after E17, and are homogeneously distributed in lamina I through V at P0 [53,57]. The density of GABAergic terminals further increases during postnatal development. In contrast, glycinergic terminals are absent in lamina I to IV during embryonic development. During postnatal development, GlyT2 is localized at GABAergic axon terminals, as detected in the ventral horn. Thus, GABAergic terminals gradually shift to coreleasing terminals in lamina IV during the first postnatal week and lamina III during the second postnatal week. For two weeks after birth, coreleasing terminals increase in density in lamina III to V, but many GABAergic terminals remain in lamina I and II until maturation is complete.

The development processes of inhibitory terminal formation and maturation are summarized schematically in Figure 4. First, GABAergic terminals are produced by GAD expression (Figure 4D,E,H). In lamina I and II, many GABAergic neurons and terminals remain (Figure 4D). In lamina III to IX, many GABAergic terminals convert to coreleasing terminals via the expression of GlyT2 (Figure 4F,I). After the initiation of high-affinity glycine uptake, both synthesized GABA and uptaken glycine are loaded into the same synaptic vesicles and coreleased from these terminals (Figure 4G,J). In lamina III to VI, coreleasing neurons and terminals are dominant (Figure 4G). In lamina VII to IX (motor neuron pools) of the ventral horn, many coreleasing terminals become glycinergic via the disappearance of GAD (Figure 4K). The mechanism underlying regional differences in the differentiation of terminals is still unclear. Furthermore, this developmental shift in inhibitory neurotransmitters from GABA to glycine has been reported previously in electrophysiological experiments in the spinal cord [108,109] and other regions [110,111,112,113].

### 3.4. Developmental Formation of Total Inhibitory Terminals

As VGAT/VIAAT transports not only GABA but also glycine into synaptic vesicles, VGAT immunohistochemistry allows for the visualization of all types of inhibitory terminals, including GABAergic, coreleasing, and glycinergic terminals. Inhibitory terminals appeared in the marginal zone at E11 and ventral horn at E13 [57]. The developmental expression of VGAT exhibits ventral-to-dorsal gradation (Figure 4), which indicates that inhibitory terminals are gradually formed in the ventral–dorsal direction during embryonic and early postnatal development [53,57].

## 4. Developmental Changes in Ionotropic GABA and Glycine Receptors

### 4.1. GABA_A_ Receptor

The subunit composition of GABA_A_ receptors changes during spinal cord development [114,115], as in various other brain regions [20,115]. In the rat spinal cord, GABA_A_ receptors first appear in the ventricular zone. The main subunit composition continued to be α4β1γ1. These receptors in the ventricular zone may be independent of synaptic transmission [116] because synapses have not yet been formed within the ventricular zone. In the gray matter, developing neurons start to express the α2, α3, α5, β2, β3, γ2, and γ3 subunits. The α1 subunit appears after birth, but the expression is low to moderate. The α2 subunit continues to be highly expressed in the developing and mature motor neurons in lamina VIII and IX. The expression of the α2 subunit gradually increases in other laminae during embryonic development but decreases after birth. Expression of the α3 and α5 subunits begins homogeneously and increases in intensity during late embryonic and early postnatal development. After birth, α3 expression decreases in the ventral horn but remains high in the dorsal horn. The α5 subunit also decreases in expression throughout the gray matter, but expression remains moderate in the motor neurons. Expression of the β2 and γ3 subunits declines during postnatal development and is low or faint in the mature spinal cord. In contrast, the expression level of the β3 and γ2 subunits continues to increase after birth and remains high [114,115]. The developmental changes in α2, α3, β3, and γ2 subunit expression parallel the change in the formation of GABAergic synapses. In the mature spinal cord, expression of the α2 subunit is high in motor neurons and the surrounding satellite neurons in the ventral horn. The α3 subunit is highly expressed in the dorsal horn and moderately localized in other regions of gray matter. In contrast, expression of the α1 and α5 subunits is weak, whereas the β3 and γ2 subunits are highly expressed throughout the gray matter.

### 4.2. GABA_B_ Receptor

In the ventricular zone, only the B1 subunit is expressed [117]. In the gray matter, both B1 (1a and 1b) and B2 subunits are highly expressed during the late embryonic and early postnatal period [75]. During postnatal development, both expressions slightly decreased, as shown by [3H]-baclofen binding assay [74].

### 4.3. GABA_C_ Receptor

After birth, the GABA_C_ receptor ρ1 subunit continued to be expressed throughout the gray matter neurons. In contrast, the ρ2 subunit continued to be expressed in the motor neurons and associated interneurons [76]. Therefore, GABA_C_ receptor subunit composition does not change during postnatal spinal cord development.

### 4.4. Glycine Receptors

The subunit combination [72,118] and electrophysiological characteristics [51,119,120,121] of glycine receptors also markedly change during spinal cord development. During embryonic development, the α2 subunit is highly and exclusively expressed throughout the gray matter of the rat spinal cord. Around the day of birth, expression of the β and α1 subunits is initiated. After birth, α2 subunit expression gradually decreases, whereas α1 and β subunit expression continue to increase. During postnatal development, the expression of the α3 subunit slightly increases [122]. In the mature spinal cord, α2 and α3 expressions are low. These results suggest that the glycine receptor exists as an α2 homomeric pentamer during embryonic development and may temporally change to an α2β heteromeric receptor. Ultimately, the major composition of this receptor changes to α1β during postnatal development [50]. In addition, those containing the α2 and α3 subunits may play roles at the extra-synaptic region in the dorsal horn, as seen in the hippocampus [123]. These changes in composition agree with the results of in vitro electrophysiological studies [120,121].

### 4.5. Developmental Formation of GABA and Glycine Removal System

GABA is removed explicitly by GAT-1 and GAT-3 [35,124,125], and glycine is removed by GlyT1 and GlyT2 during CNS development and in the mature stage (Figure 1A,B) [52]. GAT-1 and GlyT2 are localized at distinct axon terminals, while GAT-3 and GlyT1 are localized on the astrocytic sheets that face the synaptic cleft [53,57].

### 4.6. Uptake into the Presynaptic Terminals

In the marginal zone and dorsal horn, the GABA removal system at the presynaptic terminals develops simultaneously with the formation of the presynaptic terminals [77]. First, GAT-1 is localized at the GABAergic axon terminals in the marginal zone. In the dorsal horn, GAT-1 localization begins in the axon terminals at E15, and the GAT-1-positive terminals markedly increase in density during embryonic and early postnatal development [77]. During dorsal horn development, localization of GATs spreads in the deep-to-superficial direction, as is observed in GABAergic terminal formation [57]. In the ventral horn, however, although numerous GABAergic terminals and synapses temporally function during embryonic and early postnatal development, GAT-1 immunolabeling is always sparse. These results suggest that the presynaptic GABA removal system does not function at the temporal GABAergic synapses in the ventral horn, and GABA, which is released from transient terminals, may be largely removed into astrocytes near these temporal GABAergic synapses. In summary, the GABA removal system develops simultaneously with the formation of “permanent” presynaptic terminals in the spinal cord [77].

The onset of the GlyT2 expression was almost concomitant to the initial distribution of glycine immunolabeling [53,62,96]. As mentioned in Section 2.2, uptake from the extracellular space through GlyT2 is the main glycine supply pathway, suggesting that GlyT2 localization at the presynaptic terminals may mark the common onset of both glycinergic transmission and glycine removal. Therefore, the development of the glycine removal system proceeds simultaneously with the formation of glycinergic transmission [53].

### 4.7. Reuptake into the Astrocytes

**Figure 5:**
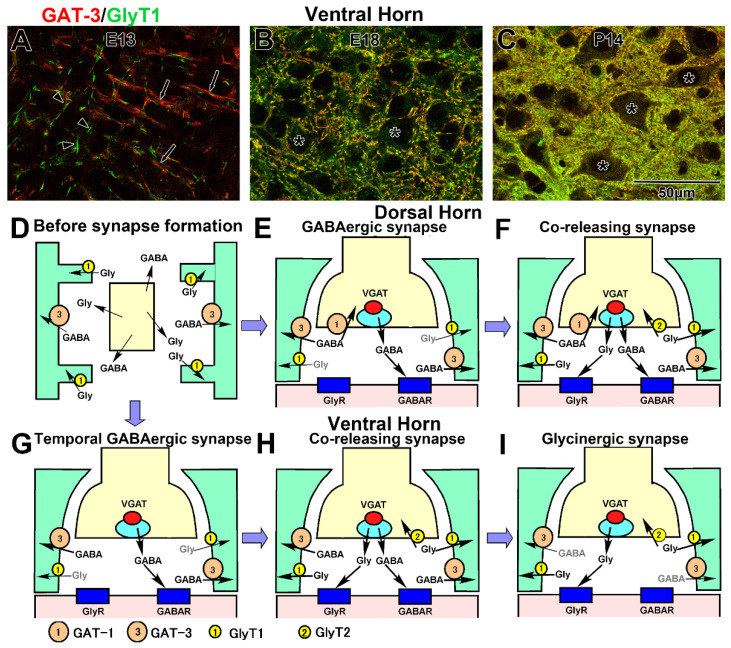
Developmental formation of the GABA and glycine removal system. (**A**–**C**) Double staining of GAT-3 (red) and GlyT1 (green). GAT-3 is localized at the shaft of the radial glial processes (arrows), whereas GlyT1 is localized at the spines of the processes (arrowheads) at E13. As development proceeds, GAT-3 and GlyT1 immunolabelings gradually merge and surround the motor neurons (asterisks in **B**,**C**). (**D**–**I**) Schematic illustrations of the development of the GABA and glycine removal system. Initially, uptake of extrasynaptically released GABA and glycine occurs at the shafts and spines of radial glial processes, respectively (**D**). In the ventral horn, GAT-1 is localized at the terminals when GABAergic synapses are formed (**E**). After the GABAergic synapses convert to coreleasing synapses, GlyT2 is additionally localized at the terminals (**F**). In contrast, in the ventral horn, GAT-1 is not localized at the terminals even though GABA is synthesized and released in the temporally synapses. After the synapses convert to coreleasing synapses, GlyT2 is also additionally localized at the terminals (**G**). Even after coreleasing synapses changed to glycinergic synapses, GAT-3 continued to be localized at the astroglial sheets (**I**).

Both GAT-3 and GlyT1 continue to localize in the astrocytic lineage cells, from radial glial to astrocytes, during spinal cord development [77]. The onset of GAT-3 localization on the radial glia is nearly concomitant with the distribution of GABAergic neurons in the ventral horn at E11 and dorsal horn at E13 before the formation of GABAergic terminals [77]. GlyT1 localization is almost concomitant with the appearance of glycinergic neurons at E13 [62]. When GlyT2-positive glycinergic terminals were detected at E15, GlyT1 had already been localized at the radial glial processes (Figure 5A). These results indicate that GABA and glycine removal systems may function before synaptic transmission and suggest that extrasynaptically released GABA and glycine may be exclusively removed into radial glial processes [126]. During spinal cord development, GAT-3- and GlyT1-expressing radial glia gradually spread to the dorsal area. Initially, GABA and glycine are removed at distinct positions on the radial glial processes. GAT-3 is localized at the shaft of radial processes, whereas GlyT1 is localized at the spine-like profiles of the shafts (Figure 5A). While radial glia differentiates into astroglia and astrocytic processes surrounding the synapses, GABAergic terminals gradually change to coreleasing terminals between E17 to P14. Concomitantly, GAT-3 and GlyT2 gradually colocalize at the astrocytic sheets that face the synaptic clefts (Figure 5B,C). Thus, in the coreleasing synapses, GABA and glycine are released into the same synaptic cleft and removed by adjacent transporters, GlyT1 and GAT-3. Development of the GABA and glycine removal system may be fixed by P21 [77].

Interestingly, although many GABAergic neurons remain GABAergic and do not convert to coreleasing neurons in lamina I and II, GlyT1 is abundantly localized in this region. In addition, after coreleasing terminals give rise to glycinergic terminals by the disappearance of GABA synthesis, GAT-3 continues to be abundantly localized at the astroglia sheets [77]. The developmental formation of the removal system is illustrated in Figure 5. During the middle embryonic stage, extrasynaptically released GABA and glycine are uptaken at the shaft and spines of radial glial processes, respectively (Figure 5D). In the dorsal horn, when GABAergic synapses are formed, GABA is removed through GAT-1 and GAT-3 (Figure 5E). Furthermore, GlyT1 is localized near GAT-3 on the astrocytic sheets and may remove extracellular glycine (Figure 5E). This type of synapse remains in lamina I and II. Next, GlyT2 appears at the presynaptic terminals, and GABAergic synapses convert to coreleasing synapses. In these synapses, GAT-3 and GlyT2 intermingle on the astrocytic sheets (Figure 5F). These synapses remain in lamina III to VII. In contrast, in lamina VIII and IX, when temporal GABAergic synapses are formed, GABA is removed through only GAT-3 (Figure 5G). After GABAergic synapses convert to coreleasing terminals, glycine starts to be removed by GlyT1 into the presynaptic terminals (Figure 5H). Even after coreleasing terminals convert to glycinergic terminals, GAT-1 persists at the astrocytic sheets (Figure 5I).

## 5. Developmental Changes in GABAergic and Glycinergic Action

In general, GABA and glycine induce depolarization of the membrane potential in the immature CNS, whereas these molecules act as inhibitory neurotransmitters in the mature CNS [79,80,127]. This developmental change in the action of these neurotransmitters occurs as a result of a negative shift in the Cl^−^ reversal potential due to the marked increase in KCC2 localization and decrease in NKCC1 expression (Figure 2A) [79,80,81]. Changes in KCC2 activity may play a pivotal role in the fine-tuning of [Cl^−^]_i_ for the following reasons [128,129,130,131]. During development, the expression of NKCC1 does not markedly change [132], whereas changes in the levels of KCC2 correlate with modification of the action of GABA. Transfection of KCC2 into hippocampal neurons converts the action of GABA from excitatory to inhibitory, and GABA is excitatory in KCC2 knockout mice [80,81,128]. Furthermore, after nerve injury, expression of KCC2 is markedly decreased in motor neurons, and the action of GABA and glycine is shifted from inhibitory to excitatory [133,134,135,136]. At E11, weak KCC2 signals appear on the surface of the ventral horn, whereas the dorsal horn is negative for KCC2 expression (Figure 2B). During embryonic development, KCC2 expression gradually increases in intensity, and the KCC2-positive area gradually spreads to the dorsal horn. The gray matter becomes homogeneously labeled by the day of birth [57,137,138]. Concomitantly, NKCC1 expression gradually decreases [138]. These results suggest that the action of GABA and glycine may change from excitation to inhibition in the direction from the ventral-to-dorsal horn [57]. As the expression of KCC2 increases and that of NKCC1 decreases, the reversal potential of Cl^−^ and inhibitory postsynaptic potentials (IPSPs) gradually decreases [132,138,139]. Taken together, these observations indicate that GABAergic and glycinergic action in the ventral horn may developmentally change as follows: initially, GABA and glycine mediate depolarization and induce action potentials; next, they mediate depolarization of the membrane potential, but the depolarization is below threshold; and finally, the excitatory inputs are shut around the birthday. This membrane potential is termed “depolarizing IPSP”, and this phenomenon is called a “shunting effect”. Finally, GABA and glycine induce hyperpolarization of the membrane potential [140,141].

The activity of KCC2 is regulated not only by expression level but also through various other mechanisms, such as phosphorylation/dephosphorylation [142,143,144] and membrane trafficking [145,146,147]. For example, phosphorylation of threonine residues 906 and 1007 decreases the activity of KCC2. In the developing CNS, this phosphorylation inhibits KCC2 activity, maintains the excitatory action of GABA and glycine, and may play key roles in morphogenesis [148]. Conversely, continuous phosphorylation affects the postnatal mouse brain functions; phosphomimetic KCC2 knock-in mice cannot survive due to the lack of GABAergic inhibition [149]. The phosphorylation of serine residue 940 increases the influx of K^+^ and Cl^−^ ions. Abnormalities in phosphorylation may cause various neuropsychiatric diseases [143,144]. The phosphorylation of tyrosine residue 1087 is involved in the internalization of KCC2, which results in the downregulation of KCC2 activity [147]. In addition to tyrosine residue phosphorylation, other complex mechanisms may take part in the trafficking and endocytosis of KCC2 and regulate the activity of KCC2 in developing neurons [145,146,147].

## 6. Discussion

Lastly, we will focus on the processes in which GABAergic excitatory action plays a role. Glycine may play a similar role in the developing CNS. It is thought that GABA may act as a trophic factor in the developing CNS and induce brain morphogenesis. In the developing immature CNS, GABA_A_ receptor-mediated depolarization activates voltage-dependent calcium channels and N-methyl-D-aspartate-type glutamate receptors and elevates cytosolic calcium ion concentration (Figure 2A) [150,151,152,153,154,155,156,157]. The elevation of cytosolic calcium may play roles in various steps in CNS development, such as (1) stop signals for cell proliferation, (2) cell migration, and (3) neuronal maturation, which includes synaptogenesis [11,14,79,80,158,159,160]. GABA acts as an antiproliferation molecule, reduces DNA synthesis in the proliferating precursor cells, and depresses the rate of cellular proliferation via the activation of GABA_A_ receptors and other GABA_A_ receptor-related molecules [116,161]. GABA modulates neuronal migration at the femtomolar (10^−^^15^ M) to micromolar (μM) level [162,163,164]. Furthermore, exposure of neurons to GABA or GABA_A_ receptor agonists induces the synthesis of neuron-specific molecules such as neuron-specific enolase and neural cell adhesion molecules, enhances the growth rate of neuronal processes, and facilitates synapse formation by inducing the expression and targeting of GABA receptor subunits [159,165,166,167,168,169,170,171,172,173,174,175,176,177,178]. Consequently, it is suggested that lack of GABA synthesis and inhibition of GABA release may cause morphological abnormalities in the CNS, including abnormalities of the spinal cord. To reveal this hypothesis, two types of knockout mice lacking GAD67 [105,106,107] and VGAT [63,179,180,181] were established. Although they have common severe phenotypes, such as omphalocele, cleft palate, hunched posture, loss of movement, and respiratory failure, and cannot survive after birth, no morphological abnormalities were detected in the CNS of these mice. Total GAD knockout mice, which lack both GAD65 and GAD67, also exhibited normal histology in the CNS [107,182]. Furthermore, KCC2-knockout mice, in which GABA and glycine continued to be excitatory, exhibited similar phenotypes [128]. These results suggest that the abnormalities detected in the three types of knockout mice may result from hyperexcitation resulting from the loss of GABAergic and glycinergic inhibition. The inhibitory action by GABA and glycine in the ventral horn may be crucial for the survival of newborn mice. Therefore, the function of GABAergic and glycinergic excitation is still unclear.

## 7. Conclusions

Initially, six groups of GABAergic neurons are derived from five domains in the ventricular zone. Each group migrates along a distinct route, settles in distinct laminae, and forms synapses. Many GABAergic neurons remain GABAergic, mainly those located in lamina I and II. In other laminae, many of these neurons convert to GABA and glycine coreleasing neurons by initiating glycine reuptake via GlyT2. In the ventral horn, many of these neurons give rise to the glycinergic network after GABA synthesis ceases. During these developmental processes, the subunit compositions and electrophysiological characteristics of GABA and glycine receptors change. During changes in neuronal types, GABA and glycine removal systems mature. Furthermore, the action of GABA and glycine shifts from excitatory to inhibitory.

## Data Availability

Not applicable.

## Abbreviations

AF	anterior funiculus
CNS	central nervous system
[Cl^−^]_i_	intracellular chloride ion concentration
CNS	central nervous system
DH	dorsal horn
DRG	dorsal root ganglion
dI1–6, dIL, dIL_A_, dIL_B,_ MN, V0–3, V0_D_, V0_V_, V2a, and V2b	neuronal groups, classes, derived from their distinct domains of spinal cord ventricular zone
dP1–dP6, vMN, and vP0–vP3	domains of the ventricular zone in embryonic spinal cord
E	embryonic day
FP	floor plate
GABA	γ-aminobutyric acid
GAD	glutamic acid decarboxylase
GAT-1, GAT-2, and GAT-3	GABA transporter 1, 2, and 3
Gly	glycine
GlyT1 and GlyT2	glycine transporter 1 and 2
GFP	green fluorescence protein
IPSP	inhibitory postsynaptic potential
KCC2	potassium (K^+^), chloride (Cl^−^) cotransporter 2
LF	lateral funiculus
NKCC1	sodium (Na^+^)-K^+^-2Cl^−^ cotransporter 1
P	postnatal day
PF	posterior funiculus
R	GABA_A_ and glycine receptor
RP	roof plate
SHMT	serine hydroxy-methyltransferase
VDCC	voltage-dependent calcium channel
VIAAT	vesicular inhibitory amino acid transporter
VGAT	vesicular GABA transporter
VH	ventral horn
VZ	ventricular zone
I-IX	laminar number of the gray matter
1	GAT-1
3	GAT-3
